# Pathophysiology of Hemophilic Arthropathy

**DOI:** 10.3390/jcm6070063

**Published:** 2017-06-25

**Authors:** Daniela Melchiorre, Mirko Manetti, Marco Matucci-Cerinic

**Affiliations:** 1 Section of Internal Medicine, Department of Experimental and Clinical Medicine, University of Florence, 50134 Florence, Italy; marco.matuccicerinic@unifi.it; 2 Department of Geriatric Medicine, Division of Rheumatology, Azienda Ospedaliero-Universitaria Careggi, 50134 Florence, Italy; 3 Section of Anatomy and Histology, Department of Experimental and Clinical Medicine, University of Florence, 50134 Florence, Italy; mirko.manetti@unifi.it

**Keywords:** hemophilia, hemarthrosis, hemophilic arthropathy, synovitis

## Abstract

Spontaneous joint bleeding and repeated hemarthroses lead to hemophilic arthropathy—a debilitating disease with a significant negative impact on mobility and quality of life. Iron, cytokines, and angiogenic growth factors play a pivotal role in the onset of the inflammatory process that involves the synovial tissue, articular cartilage, and subchondral bone, with early damages and molecular changes determining the perpetuation of a chronic inflammatory condition. Synovitis is one of the earliest complications of hemarthrosis, and is characterized by synovial hypertrophy, migration of inflammatory cells, and a high degree of neo-angiogenesis with subsequent bleeding. The pathogenic mechanisms and molecular pathways by which blood in the joint cavity causes articular cartilage and subchondral bone destruction have yet to be fully elucidated. Both cytokines and matrix metalloproteinases and hydroxyl radicals may induce chondrocyte apoptosis. Members of the tumor necrosis factor receptor superfamily (such as the molecular triad: osteoprotegerin—OPG; receptor activator of nuclear factor κB—RANK; RANK ligand—RANKL) seem instead to play a major role in the inflammatory process. These pathogenic processes interact with each other and ultimately lead to a fibrotic joint and the disabling condition characteristic of hemophilic arthropathy.

## 1. Introduction

Hemophilia A and B are X-linked congenital bleeding disorders caused by the absence or decrease of clotting factor VIII (FVIII) or factor IX (FIX), respectively. In patients with severe hemophilia (i.e., plasma FVIII or FIX < 1 U/dL), joint bleeding is the most frequent manifestation in both children and in adults [1,2]. The benefits of the prophylactic administration of clotting factor concentrates (CFCs) to prevent the arthropathy have been defined. CFCs can be used as primary prophylaxis in the absence of documented osteochondral joint disease, before the second clinically evident joint bleeding, and with age of 3 years. Instead, they can be used as secondary prophylaxis after two or more bleedings into target joints and before the onset of clinically documented joint disease, as well as tertiary prophylaxis in the presence of overt joint disease. It is noteworthy that the use of CFCs can reduce the risk of hemarthrosis, even if the risk of bleedings is not completely prevented [3]. Recurrent joint bleedings induce joint damage in about eighty percent of the patients, with knees, elbows, and ankles as preferential target joints. 

Hemophilic arthropathy is a disabling condition characterized by joint impairment, chronic pain, and reduced quality of life [4,5]. Recent evidence indicates that intra-articular inflammation and angiogenesis may be pivotal processes in the pathogenic cascade of hemophilic arthropathy [6]. Joint bleeding favors iron release from hemoglobin, thus inducing a chronic inflammatory process mediated by cytokines and pro-angiogenic factors and consequently leading to progressive synovial pannus growth and articular cartilage damage [7,8,9,10,11]. Interestingly, a close relationship between the type of gene mutation and the clinical phenotype of hemophilia has been demonstrated. In fact, it appears that “null” mutations and/or missense mutations may significantly affect the severity of hemophilic arthropathy either in patients with hemophilia A or in those with hemophilia B [12].

## 2. Pathophysiology of Hemophilic Arthropathy

The pathophysiology of hemophilic arthropathy shares some clinical and biological features with rheumatoid arthritis (RA) and osteoarthritis (OA), especially RA-related synovitis and bone resorption and OA-related articular cartilage degeneration.

### 2.1. From Synovitis to Articular Cartilage Damage

In hemophilic arthropathy, both inflammatory and degenerative mechanisms contribute to articular cartilage degeneration, although these processes may even occur independently. Intra-articular blood effusion-derived hemosiderin deposits are thought to be critical in the early phase of hemophilic arthropathy through the induction of a proliferative disorder with chronic synovitis which then extends to the articular cartilage surface, ultimately resulting in destructive arthropathy [13,14,15,16]. However, according to other authors, the initial pathogenic process might rather involve articular cartilage damage due to the iron-catalyzed formation of destructive oxygen metabolites resulting in chondrocyte apoptosis, with synovial inflammatory changes being secondary or parallel to the process of cartilage damage [13].

**Figure 1:**
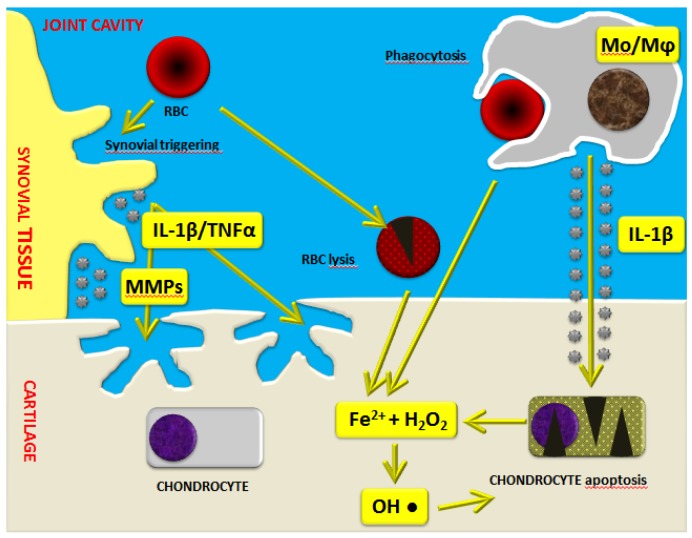
Schematic representation of the mechanisms of blood-induced joint damage in hemophilia. The role of iron (Fe^2+^) interacting with hydrogen peroxide (H_2_O_2_), activation of macrophages (Mo/Mφ), matrix metalloproteinases (MMPs), and pro-inflammatory cytokines is highlighted. Adapted with permission from [17]. IL: interleukin; RBC: red blood cell.

Recent studies point toward the implication of a high molecular weight complex in the hemophilic arthropathy-related inflammatory process. Indeed, the so-called “inflammasome” has been highlighted as a crucial factor which regulates the maturation and secretion of pro-inflammatory interleukin (IL)-1β. Iron also seems to play a crucial role in the induction of the expression of several pro-inflammatory cytokines, including IL-1α, IL-6, and tumor necrosis factor (TNF)-α (Figure 1) [17]. Moreover, it appears that iron is involved in the initiation of synovial pannus growth by dysregulating the expression of critical genes such as *c-myc* and *mdm2*, which are responsible for synoviocyte proliferation [18,19,20,21]. Synovitis is an inflammatory process involving synovial tissue, characterized by hypertrophy, migration of inflammatory cells, and a high degree of neo-angiogenesis. In addition, IL-1α, IL-6, IL-1β, and TNF-α activate monocytes/macrophages, triggering a catabolic program with the production of nitric oxide (NO), proteases such as matrix metalloproteinases (MMPs), tissue plasminogen activator, and other matrix components [19,22,23]. These in turn can act on T cells, fibroblasts, and osteoclasts through a variety of inflammatory mediators, leading to articular cartilage and subchondral bone destruction. 

As demonstrated by Jansen et al. [24], monocytes/macrophages are mostly responsible for the irreversible inhibition of cartilage matrix synthesis and the induction of chondrocyte apoptosis. In particular, elevated levels of IL-1β produced by activated monocytes/macrophages further increase the release of hydrogen peroxide by chondrocytes with the formation of cytotoxic hydroxyl radicals sited very close to the chondrocytes (Figure 1) [17,19]. Moreover, there are also direct harmful effects exerted by intra-articular blood on cartilage, as demonstrated by in vitro studies. Indeed, it has been reported that a short four-day exposure of human cartilage to whole blood at concentrations up to 50% may induce long-lasting inhibition of cartilage matrix proteoglycan synthesis and a prolonged decrease in proteoglycan content [25,26,27,28,29].

In this complex scenario, the role of neo-angiogenesis also deserves careful consideration. In fact, neo-angiogenesis—a process implicated in tumor growth and inflammatory arthritis—is also a critical independent mechanism involved in hemophilic arthropathy [15]. Pro-angiogenic vascular endothelial growth factor (VEGF) is a pivotal signaling molecule involved in angiogenesis, can be induced by hypoxia and some cytokines, and acts mainly through the interaction with its receptors, VEGFR-1 and VEGFR-2 [6,13,16]. Studies on the formation of synovial pannus in other joint diseases reported an increased oxygen demand with evidence of de novo blood vessel formation in the synovium [11,30]. In patients with severe hemophilia, either elevated circulating levels of VEGF-A or increased synovial expression of VEGF-A have been reported, which suggests an important role of this powerful pro-angiogenic mediator in the pathogenesis of hemophilic arthropathy-related synovitis [6,31]. Interestingly, a direct correlation between high serum VEGF-A levels and disease activity has also been demonstrated in RA [7,10]. In particular, increased microvessel density and the expression of VEGF have been shown in the synovium of severe hemophilic arthropathy patients with advanced joint disease [32]. These data indicate that the advanced stage of hemophilic arthropathy is characterized by active angiogenesis.

### 2.2. Bone Damage

The mechanisms by which the bleeding in hemophilic arthropathy leads to subchondral bone damage and loss are not yet clear. In hemophilic arthropathy patients, osteoporosis characterized by bone loss was often related to the presence of infectious comorbidities and their treatment. However, it was shown that osteoporosis in patients with hemophilic arthropathy may even be independent of the aforementioned factors [33].

Even though some cytokines have been related to the pathogenesis of synovitis in hemophilic arthropathy with some similarities to RA, few studies have clearly demonstrated their functional role in the pathogenic cascade of the disease [18]. In fact, hemophilic arthropathy has been firstly described as a degenerative rather than an inflammatory joint disease [34]. However, recent studies indicate that hemophilic arthropathy has similarities either with the degenerative joint damage occuring in OA or with the chronic inflammatory process associated with RA, even if specific pathogenetic mechanisms have not yet been fully elucidated [35].

**Figure 2:**
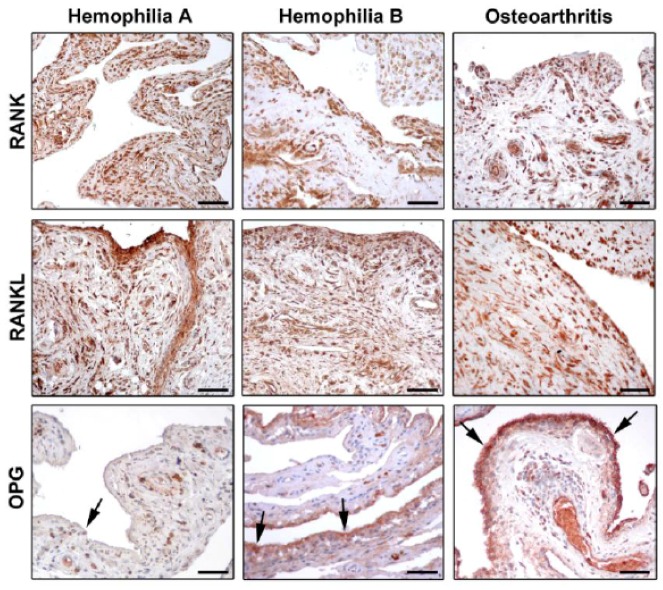
Photomicrographs showing the expression of receptor activator of nuclear factor-κB (RANK), RANK ligand (RANKL), and osteoprotegerin (OPG) in synovial tissue from patients with hemophilia A, hemophilia B, and osteoarthritis. Representative photomicrographs of tissue sections subjected to immunoperoxidase staining for RANK, RANKL, and OPG (brownish-red color) and counterstained with hematoxylin are shown. Arrows indicate OPG immunostaining in the synovial lining layer. Original magnification: ×20. Scale bar: 100 µm. Reproduced with permission from [46].

A crucial regulator of bone biology is the molecular triad osteoprotegerin (OPG)/receptor activator of nuclear factor κB (RANK)/RANK ligand (RANKL) [36,37], which controls the local changes in bone turnover and represents a key pathway triggering bone resorption induced by inflammation. RANKL is a transmembrane ligand mainly expressed on osteoblasts/stromal cells in the bone microenvironment. It is synthesized by lymphocytes and synovial cells, and may induce osteoclastogenesis through a mechanism enhanced by several cytokines (e.g., TNF-α, IL-1, and IL-17) that promote both inflammation and bone resorption [38,39]. By binding to its receptor RANK which is expressed on the cell surface of osteoclast precursors, RANKL induces osteoclast differentiation and maturation, thus fostering bone resorption. In this scenario, OPG acts as a decoy receptor for RANKL, and competes for the binding of RANKL to RANK [40,41,42]. By this mechanism, OPG negatively regulates osteoclast differentiation, activity, and survival both in vivo and in vitro, thus effectively inhibiting bone resorption [37,39,42]. Accordingly, any change in the balance between OPG and RANKL may lead to pathological bone remodeling. A high prevalence of osteoporosis has been reported among hemophiliacs, which is related to the severity of arthropathy and is enhanced by HIV infection. Therefore, increased bone resorption does not seem to be balanced by a comparable bone formation in these patients [43,44]. Of note, the triad OPG/RANK/RANKL may act as key regulator of bone remodeling in synovial tissue of adult hemophiliacs, as suggested by decreased OPG levels and the strong expression of RANK and RANKL (Figure 2) [45,46]. Moreover, a strict correlation between the severity of hemophilic arthropathy and instrumental findings, such as the World Federation of Hemophilia (WFH) orthopaedic joint scale [47], Petterson [48], and ultrasound (US) [49] scores, has been reported [45]. The molecular markers of bone turnover in the synovial tissue of hemophiliacs clearly indicate an osteoclastic activation which is not counteracted by OPG [45]. In fact, RANK and RANKL were found to be strongly expressed in the synovium, independently of the type of treatment. Instead, the expression of OPG was dramatically reduced in the synovial tissue of hemophilic arthropathy patients [45]. The almost complete absence of OPG expression in hemophilic arthropathy synovial tissue represents an important issue, because it implies that the bone turnover balance is shifted toward osteoclastic activity, and thus bone resorption. Furthermore, there is in vitro evidence that the reduction of thrombin production results in less thrombin-induced PAR-1 mediated proliferation of osteoblasts [3]. Collectively, these findings indicate that bone damage in hemophilic arthropathy is a complex multifactorial process.

## 3. Evidence That Arthropathy May be More Severe in Hemophilia A than in Hemophilia B

Recent investigations suggest that patients with severe hemophilia B may have a less-severe disease compared to patients with severe hemophilia A [12,50,51]. In a recent study, a large cohort of patients with hemophilia A and B was evaluated by employing clinical, imaging, and biochemical markers [46]. A significantly worse WFH score and US score were observed in hemophilia A patients compared with hemophilia B patients [46]. Indeed, the US score showed lower mean values for the hemophilia B patient group compared to the hemophilia A patient group. Furthermore, the evidence of lower values of WFH score in the hemophilia B patient group has strengthened the sonographic result. Interestingly, most of the US findings (e.g., effusion, synovial hypertrophy with flags on power Doppler, hemosiderin deposition, bone remodeling, cartilage modifications) were mainly observed in the hemophilia A patient group. Moreover, the correlation between the number of hemarthroses and the severity of the disease has been investigated. The results showed a greater percentage of hemarthroses in the hemophilia A patient group than in the hemophilia B patient group, even when matched for age. In addition, possible differences in the RANK/RANKL/OPG molecular triad between hemophilia A and B patients were analyzed [46]. A lower expression of OPG was found in the synovial tissue of patients with hemophilia A compared with hemophilia B, which further highlight the greater severity of arthropathy in the hemophilia A patient group (Figure 2). This evidence was further confirmed by the analysis of serum OPG levels, which resulted significantly lower in patients with hemophilia A than in patients with hemophilia B [46].

## 4. Conclusions

In summary, although the complex molecular mechanisms underlying the abnormal synovial overgrowth in hemophilic arthropathy are still poorly understood, recent studies have demonstrated that a variety of mediators may play an important role in blood-induced joint damage. Collectively, such mediators are believed to trigger a synovial over-reaction which, once started, may act independently of the intra-articular bleeding. Indeed, hemarthrosis leads to intra-articular iron deposition, synovial proliferation and neo-angiogenesis, and cartilage and subchondral bone damage, triggering a vicious cycle resulting in severe arthropathy. Although bone damage may arise from a multifactorial process in patients with hemophilia, hemarthrosis seems to be a major contributor. This complex scenario ultimately leads to the clinical manifestation of hemophilic arthropathy, which has a more severe phenotype in hemophilia A than in hemophilia B patients.
